# The Effect of Curcumin on Lipid Profile and Glycemic Status of Patients with Type 2 Diabetes Mellitus: A Systematic Review and Meta-Analysis

**DOI:** 10.1155/2022/8278744

**Published:** 2022-06-17

**Authors:** Jiao Tian, Bin Feng, Zhen Tian

**Affiliations:** ^1^Department of Infection, Ministry of Education Key Laboratory of Child Development and Disorders, National Clinical Research Center for Child Health and Disorders, China International Science and Technology Cooperation Base of Child Development and Critical Disorders, Chongqing Key Laboratory of Child Health and Nutrition, Children's Hospital of Chongqing Medical University, Chongqing 400014, China; ^2^State Key Laboratory of Military Stomatology, National Clinical Research Center for Oral Diseases, Shaanxi International Joint Research Center for Oral Diseases, Department of Pharmacy, School of Stomatology, Fourth Military Medical University, Xi'an 710032, China; ^3^College of Pharmaceutical Sciences, Southwest University, Chongqing 400716, China; ^4^Department of Pharmacy, Tangdu Hospital, Fourth Military Medical University, Xi'an 710038, China

## Abstract

Type 2 diabetes mellitus (T2DM) is a progressive metabolic disorder, some natural compounds are thought to be beneficial in improving the metabolic status of patients with T2DM. Curcumin is the main bioactive agent of turmeric, the impact of curcumin on T2DM is still controversial. This meta-analysis aimed to evaluate the effects of curcumin on lipids profile and glucose status in patients with T2DM. Randomized controlled trials (RCTs) examining the effects of curcumin on lipids profile and glycemic control of T2DM patients were searched in PubMed, Embase, Web of Science and Cochrane Library. Pooled estimates of weighted mean difference (WMD) were calculated between intervention and control groups using random-effects or fixed-effects model. Subgroup and sensitivity analyses were conducted to assess the effects. Nine eligible RCT with 604 subjects were included. The estimated pooled mean changes with curcumin were −18.97 mg/dL (95% CI: −36.47 to −1.47; *P*=0.03) for triglyceride (TG), −8.91 mg/dL (95% CI: −14.18 to −3.63, *P*=0.001) for total cholesterol (TC), -4.01 mg/dL (95% CI: -10.96 to 2.95, *P*=0.259) for low density lipoprotein cholesterol (LDL-c), 0.32 mg/dL (95% CI: −0.74 to 1.37, *P*=0.557) for high density lipoprotein cholesterol (HDL-c), −8.85 mg/dL (95% CI: −14.4 to −3.29, *P*=0.002) for fasting blood glucose (FBG), -0.54 (95% CI: −0.81 to −0.27, *P* ≤ 0.001) for glycated hemoglobin (HbA1c) (%) compared with controls. There was a significant heterogeneity for the influence of curcumin on TG, LDL-c, FBG and HbA1c. Subgroup analysis revealed that the heterogeneity mainly attributed to trial period, curcumin dosage and other therapy. The results of this study showed that curcumin supplementation had beneficial effects on glycemic status and some lipid parameters in patients with T2DM. Further studies with large-scale are still needed to confirm the results.

## 1. Introduction

As one of the most common metabolic disorders, diabetes mellitus brings a huge burden to public health worldwide. According to a report of the International Diabetes Federation (IDF), the people diagnosed with diabetes were 537 million in 2021, and this ratio is still on the rise [1]. Dyslipidemia is a common comorbidity of type 2 diabetes mellitus (T2DM), which is characterized by elevated triglyceride (TG), total cholesterol (TC), low-density lipoprotein cholesterol (LDL-c) level, and/or decreased high-density lipoprotein cholesterol (HDL-c) concentration in serum [2]. Dyslipidemia and dysglycemia interact with each other, and they are the main risk factors of macro- and microvascular diseases in T2DM. Without effective interventions, they will lead to atherosclerosis and cardiovascular disease (CVD) progressively. Although some effective intervention strategies have been formulated for improving glycemic status of T2DM patients, they often need lipid-lowering drugs at the same time to prevent CVD. This combination of drugs not only increases the economic burden but also aggravates side effects. For example, statin therapy is the fundamental measure to treat dyslipidemia in T2DM, whereas it increases the risk of new-onset diabetes and myopathy [3]. The current situation suggests that novel therapeutic interventions are needed to manage dyslipidemia and dysglycemia in diabetic patients.

Natural products and dietary agents have gained more and more attention over the years. Compared with synthetic drugs, they have a good safety profile because of their natural properties. *Curcuma longa* L. is a traditional medicinal plant, which is widely distributed in China and some Asian countries. Turmeric, the rhizome of *Curcuma longa* L, is used as a spice to improve taste and also as medicine because of its therapeutic properties [4]. Curcuminoids, the main bioactive agents extracted from the rhizome of *Curcuma longa* L, is responsible for the major biological effects of turmeric [5]. As the main present form of curcuminoids, curcumin has a wide range of pharmacological effects, including antioxidant, anti-inflammatory, antibacterial, antiviral, antifungal, and antitumor properties [6–8]. Experimental and clinical studies also have reported the beneficial effects of curcumin supplementation on lipid profile [9, 10] and glycemic status [11]. However, the results of clinical trials are controversial. Some studies have shown favorable effects of curcumin [11,12], whereas others have failed to report promising effects [13,14]. For example, in a 12-week clinical trial, curcuminoids significantly reduced serum TC level and increased HDL-c level in patients with T2DM but had no significant effect on serum TG and LDL-c concentrations [12]. Another study reported that taking curcuminoids for three months only resulted in a significant decrease in serum TG but had no remarkable effect on the level of TC, LDL-c, and HDL-c of overweight T2DM patients [15].

As a phytomedicine contained in dietary supplementation, clarifying the effects of curcunin is important because it is helpful for assessing its potential as an alternative and complementary medicine on improving the metabolic status of T2DM patients. Thus, a meta-analysis of published clinical trials about curcumin is necessary. It can offer a more accurate and precise estimate of the overall effect of curcumin on T2DM patients through overcoming the limitations of small sample sizes and increasing the statistical power. Therefore, we conducted this meta-analysis of all published randomized controlled trials and synthesized the available data qualitatively and quantitatively in order to outline curcumin's efficacy and possible uses in clinical practice.

## 2. Methods

This study was performed following the Preferred Reporting Items for Systematic Reviews and Meta-Analyses (PRISMA) 2020 checklist [16].

### 2.1. Search Strategy

A comprehensive and systematic literature search was conducted on articles published in English from inception through March 2022 in multiple electronic databases: Pubmed, Embase, Web of Sciences, and the Cochrane Library. The search strategy aimed to evaluate the effects of curcumin on patients with T2DM, so the following combination of key words and MeSH terms related to the intervention were used: (curcumin OR curcuminoid OR turmeric OR curcuma OR Meriva) AND (diabetes OR diabetic). To ensure a comprehensive identification of additional potential publications, the reference lists of related trials were also detected. The scope of the search was limited to human studies.

### 2.2. Eligibility Criteria

A literature management software (EndNote X9; Thomson Reuters, New York) was used to accelerate the process of screening citations. The duplicates were removed automatically when the search results were imported into EndNote X9. Then the remaining articles were screened by two independent authors (JT and BF) based on the titles and abstracts. The full-texts of selected articles were included in this meta-analysis if they met the following inclusion criteria: 1) human RCTs with parallel or crossover design, 2) the research participants should be Type 2 diabetic patients over 18 years, 3) curcumin or combined curcuminoids, turmeric extract, or turmeric powder in the intervention group and placebo or medication in the control group (if the control group was a medication, then the intervention group was treated with curcumin and this medication), 4) adequate information was accessible, including the baseline and endpoint values or net changes between the two points with mean, SD, SE, number of participants, or 95% CIs for the intervention and control groups. The studies were excluded if they met anyone of the following criteria: (1) uncontrolled trial, (2) including participants who were not T2DM, (3) including participants who were younger than 18 years, and (4) studies did not report sufficient data.

### 2.3. Data Extraction

Data extraction was performed independently by two investigators according to inclusion criteria. The following information of eligible studies was extracted: first author's name, year of publication, study design, study group, sample size, treatment duration, treatment dosage, the baseline, endpoint values or net changes of lipid (TG, TC, LDL-c, and HDL-c), and glycemic parameters (FBG and HbA1c). Inconsistencies between the two investigators regarding the eligibility of studies were resolved by discussion or with the assistance of a third investigator.

### 2.4. Quality Assessment

The quality assessment of selected studies was conducted independently by two investigators. The risk of bias was evaluated according to th*e Cochrane Handbook for Systematic Reviews* from the following aspects: adequate sequence generation, allocation concealment, blinding of the participants and personnel, blinding of outcome assessment, incomplete outcome data, selective outcome reporting, and other sources of bias.

### 2.5. Data Synthesis and Statistical Analysis

All analyses were conducted with the software STATA version 12.0 (Stata Corp, College Station, TX). The mean, SD, and *n* information about the net change of lipid profiles (TG, TC, LDL-c, and HDL-c) and glycemic control (FBG, HbA1c) in both the intervention and control groups were collected. The means and SDs of net change were calculated if the trial did not report them directly: mean_change_ =mean_final_ − mean_baseline_, SD_change_ = square root ((SD _baseline_)^2^ +(SD_final_)^2^− 2R × SD _baseline_×D_final_), assuming a correlation coefficient (*R*)= 0.5 [17]. All values of lipid and glycemic parameters were collated as mg/dL, using a conversion factor of 38.66 (for cholesterol), 88.6 (for triglycerides), and 18 (for glucose) to change from mmol/L to mg/dL if necessary. The difference in net changes of glycemic status and lipid profiles between the intervention and control groups was taken as the effect size. The effect size of curcumin on glycemic status and lipid profiles for both groups wascalculated from the weighted mean difference (WMD) with 95% confidence intervals (CI) using the DerSimonian and Laird random-effects model or inverse-variance fixed-effects model.

Heterogeneity was quantitatively evaluated by using the *I*^*2*^ statistic. We considered *I*^*2*^ > 30% to indicate significant heterogeneity between the trials [18]. A random-effect model was used to pool the data in the presence of significant heterogeneity among studies; otherwise, a fixed-effect model was adopted [19]. Sensitivity analysis and subgroup analysis were performed to determine the source of heterogeneity if *I*^*2*^ > 30%. A sensitivity analysis was conducted to examine the influence of each study on the overall effect size. Subgroup analysis was conducted based on the following potential moderator variables: duration of study, intervention dosage, and coexisting of other therapy or not. The potential publication bias was detected by Egger's test. A statistically significant difference was considered by*P* < 0.05.

## 3. Results

### 3.1. Literature Search and Characteristics of the Included Studies

**Figure 1:**
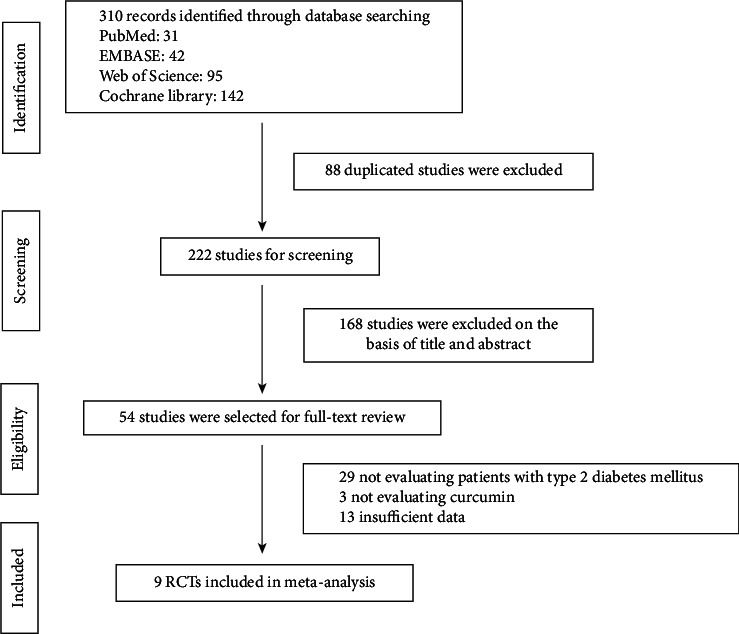
The flow summary of data search and study selection.

The multidatabase search yielded 310 studies in total, and 222 studies were screened after removing the duplicates. Of these, 168 studies were discarded after screening titles and abstracts. The remaining 54 studies were selected for full-text review, and 45 studies were excluded after eligibility assessment. Finally, nine RCTs satisfied inclusion criteria and were included in this meta-analysis [11,12,14,15,20–24]. A flow diagram for article identification and selection is presented in Figure 1.

A total of 604 participants (284 in the curcumin group and 281 in the control group) were included in the selected studies. The design of all trials was parallel; seven of them were double-blind RCTs, and the other two were open label RCTs. All subjects in these trials were patients with T2DM, and their mean age ranged from 41 to 60.95 years old. Both the doses and forms of curcumin varied among the included studies. As for the forms of agents used in the intervention group, turmeric, curcuminoids, and curcumin each accounted for one third of the nine trials. The dosage of agent supplementation in the intervention group ranged from 80 to 2100 mg/day, and it varied from 46 to 1500 mg/day if converted to standardized curcumin. The duration of intervention was between 4 weeks and 3 months in different studies. The basic characteristics of the included trials are shown in Table 1.

### 3.2. Quality Assessment

**Figure 2:**
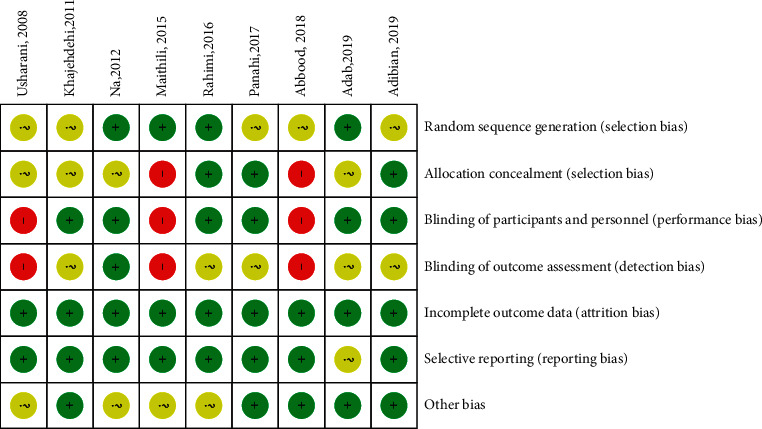
Risk of bias assessment of the included trials.

The risk of bias in the included studies is summarized in Figure 2. With respect to the random sequence generation, four RCTs were judged to have a low risk of bias and the other five studies were classified as unclear due to insufficient information. Regarding the concealment of allocation, four trials did not present enough information about the concealment procedure, and two trials showed a high risk of bias. Three trials had a high risk of bias for blinding, and five trials did not present details about blinding of outcome assessment. All of the included studies presented a low risk of bias for incomplete outcome data. Almost all included studies exhibited a low risk of bias regarding selective reporting, with only one study judged as unclear. In addition, four studies were unclear about other biases.

### 3.3. Meta-Analysis Results

#### 3.3.1. Effect of Curcumin on TG

**Figure 3:**
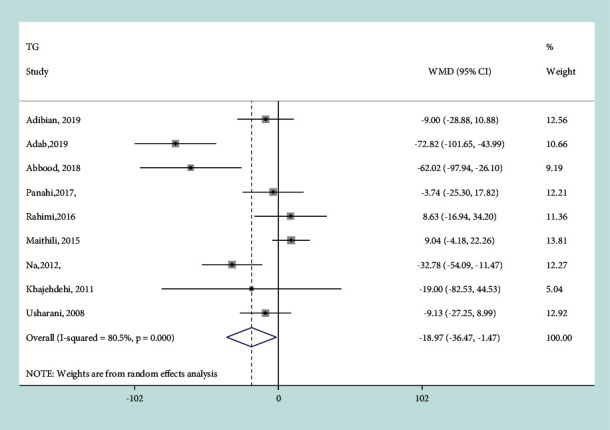
Results of meta-analysis of studies comparing curcumin to control on change from baseline in TG. Weight was assigned by STATA using the number of subjects and SD. The sizes of data markers indicated the weight of each study in the analysis. The black diamond represents the pooled-effect size.

Combining the effect sizes from nine studies based on the random-effects model, a significant difference was observed between curcumin supplementation and control treatment (WMD =− 18.97 mg/dL, 95% CI−36.47 to−1.47 mg/dL, *P*=0.03) (Figure 3). These results indicated that curcumin could reduce TG concentration significantly. However, the heterogeneity among the included trials was high (*I*^*2*^ = 80.5%, *P* ≤ 0.001). Then, subgroup analysis was performed to explore the sources of between-study heterogeneity (Table 2). Heterogeneity of TG was partly due to treatment dosage of curcumin and coexistence of other therapies or not. Greater reduction in TG levels was observed using pooled data from trials with the curcumin dose >100 mg/day (−28.944 mg/dL, 95% CI:−49.620, −8.268 mg/dL, *P*=0.006) and with the participants received other therapy (−39.449 mg/dL, 95%CI: −65.370, −13.527 mg/dL, *P*=0.003). Sensitivity analysis did not find the significant influence of each trial on the pooled effect of curcumin on TG.

#### 3.3.2. Effect of Curcumin on TC

**Figure 4:**
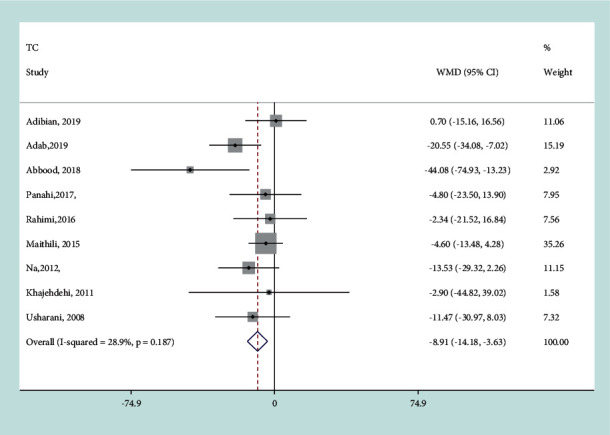
Results of meta-analysis of studies comparing curcumin to control on change from baseline in TC.

Since no heterogeneity was detected in the analysis (*I*^*2*^ = 28.9%, *P*=0.187), the fixed-effects model was used for analysis. The mean difference in net changes of TC concentrations between the intervention and control groups was −8.91 mg/dL (95% CI −14.18 to −3.63 mg/dL, *P*=0.001) (Figure 4). It suggested that curcumin supplementation significantly decreased the serum TC level of patients with T2DM.

#### 3.3.3. Effect of Curcumin on LDL-C

**Figure 5:**
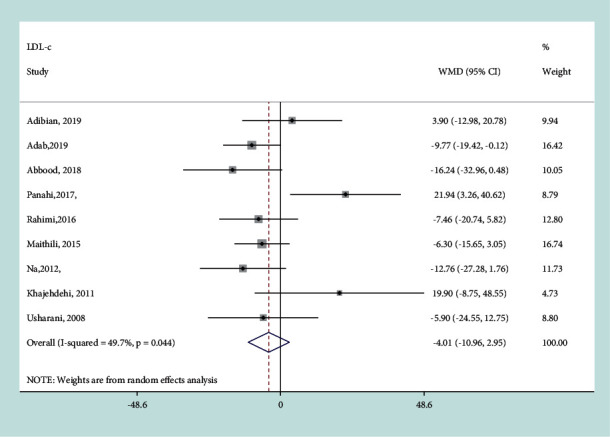
Results of meta-analysis of studies comparing curcumin to control on change from baseline in LDL-c.

The between-study heterogeneity was high (*I*^*2*^ = 49.7%, *P*=0.044), so the random-effects model was used for analysis. There was no significant difference in the net change of LDL-c between the curcumin intervention group and the control group (WMD = − 4.01 mg/dL, 95% CI: −10.96, 2.95 mg/dL, *P*=0.259) (Figure 5). Subgroup analysis revealed that treatment duration, curcumin dosage, and other therapy were the main sources of heterogeneity (Table 2). Sensitivity analysis did not find the significant influence of each trial on the pooled effect of curcumin on LDL-c.

#### 3.3.4. Effect of Curcumin on HDL-C

**Figure 6:**
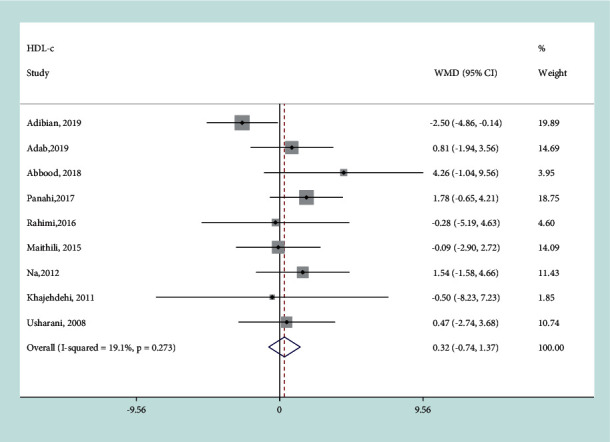
Results of meta-analysis of studies comparing curcumin to control on change from baseline in HDL-c.

There was no heterogeneity in included studies (*I*^*2*^ = 19.1%, *P*=0.273) and the fixed-effects model was used for analysis. No significant change was found in HDL-c in the curcumin intervention group compared with the control group (WMD = 0.32 mg/dL, 95% CI: −0.74, 1.37 mg/dL, *P*=0.557) (Figure 6). It indicated that curcumin supplementation may have no evident influence on the serum HDL-c of patients with T2DM.

#### 3.3.5. Effect of Curcumin on FBG

**Figure 7:**
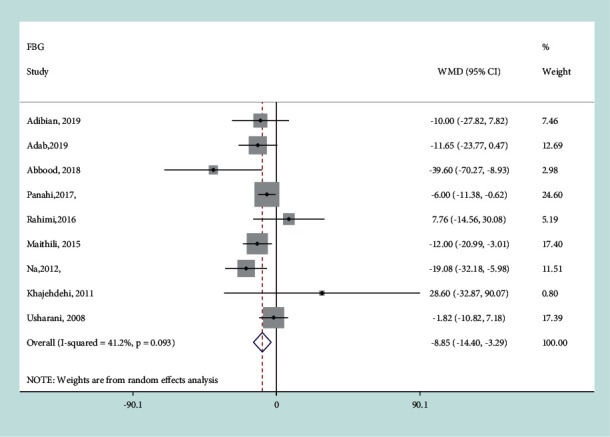
Results of meta-analysis of studies comparing curcumin to control on change from baseline in FBG.

The random-effects model was used for analysis because of the relatively high heterogeneity between the studies (*I*^*2*^ = 41.2%, *P*=0.093). Curcumin significantly reduced blood glucose levels compared with control treatment (WMD = − 8.85 mg/dL, 95% CI: −14.4, −3.29 mg/dL, *P*=0.002) (Figure 7). The subgroup analysis was performed to detect the source of heterogeneity and it was found that heterogeneity was mainly attributed to the treatment duration, curcumin dosage, and other therapy (Table 2). The effect was more powerfully significant in trials with the treatment duration >8w (−10.893 mg/dL, 95% CI:−21.149, −0.636 mg/dL, *P*=0.037), curcumin dose >100 mg/day (−9.798 mg/dL, 95% CI:−16.375, −3.221 mg/dL, *P*=0.004), and with the participants receiving the other therapy (−15.185 mg/dL, 95% CI: −24.819, −5.55 mg/dL, *P*=0.002) (Table 2). Sensitivity analysis did not find the significant influence of each trial on the pooled effect of curcumin on FBG.

#### 3.3.6. Effect of Curcumin on HbA1c

**Figure 8:**
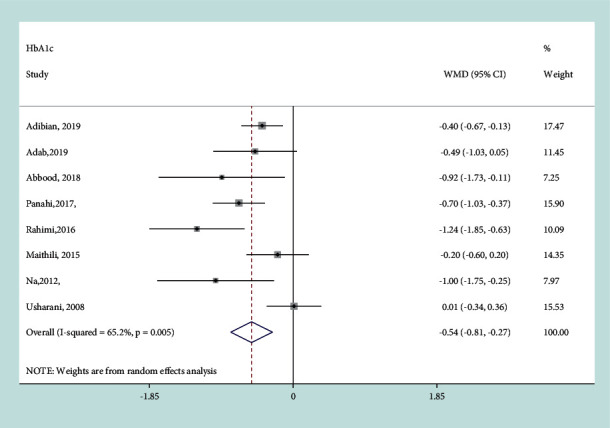
Results of meta-analysis of studies comparing curcumin to control on change from baseline in HbA1c.

There was a remarkable reduction in HbA1c (%) in the curcumin intervention group compared with the placebo-control group (WMD = − 0.54, 95% CI: −0.81, −0.27, *P* ≤ 0.001) based on the analysis with a random-effects model (*I*^*2*^ = 65.2%, *P*=0.005) (Figure 8). Subgroup analysis found that the treatment duration and other therapy were the main sources of heterogeneity (Table 2). Greater reduction in HbA1c (%) was observed in the pooled data from trials with the treatment duration >8w (−0.749, 95% CI:−1.059, −0.439, *P* ≤ 0.001). Sensitivity analyses did not alter the results.

### 3.4. Publication Bias

All data was analyzed in accordance with the intention-to-treat principle. Egger's test was used to detect the publication bias of lipid profiles and glycemic status. The results showed that there was no significant potential publication bias in examining the effects of curcumin on TG (*P*=0.091), TC (*P*=0.399), LDL-c (*P*=0.126), HDL-c (*P*=0.479), FBG (*P*=0.727) and HbA1c (*P*=0.158). Funnel plots were not used to indicate the publication bias due to an insufficient number of included studies (less than 10) [25].

## 4. Discussion

T2DM is a kind of metabolic disease characterized by impaired glucose metabolism. It reveals that the incidence of dyslipidemia is higher in patients with T2DM than in healthy ones [26]. Dyslipidemia and dysglycemia negatively interact with each other and are both the strong risk factors for CVD [27,28]. Because of the inherent shortages of the current treatments for dyslipidemia and dysglycemia, it is a good strategy to discover natural therapies to improve lipid profiles and glucose metabolism.

This meta-analysis of RCTs reported the effect of curcumin supplementation on lipid profiles and glycemic status in patients with T2DM. Eligible criteria were met by nine trials with a total of 604 participants. Pooled data showed that curcumin significantly decreased TG, TC, FBG, and HbA1c levels and also led to a reduction in LDL-c and an elevation in HDL-c concentration, although with no statistical difference.

The significant reduction of FBG (WMD = −8.85 mg/dL, 95% CI: −14.4, −3.29mg/dL, *P*=0.002) and HbA1c (WMD =−0.54, 95% CI: −0.81, −0.27, *P* ≤ 0.001) after treatment with curcumin suggested that it improved the glycemic metabolism of T2DM patients markedly. Studies have shown that curcumin could promote insulin release through inducing *β*-cell electrical activity and lower serum glucose level via decreasing the production of hepatic glucose and increasing glucose uptake [29,30]. The anti-inflammatory effects of curcumin also played a crucial role in improving glucose metabolism by suppressing the nuclear factor-kappa B pathways [31,32] and activating peroxisome proliferator activated receptor gamma (PPAR-*γ*) [32,33].

As for the effect of curcumin on lipid metabolism of T2DM patients, we demonstrated that curcumin supplementation resulted in a reduction in the level of TG (WMD = − 18.97 mg/dL, 95% CI −36.47 to −1.47 mg/dL, *P*=0.03) and TC (95% CI −14.18 to −3.63 mg/dL, *P*=0.001), and also led to a decrease in LDL-c (WMD = − 4.01 mg/dL, 95% CI: −10.96, 2.95 mg/dL, *P*=0.259) and an increase in the HDL-c level (WMD = 0.32 mg/dL, 95% CI: −0.74, 1.37 mg/dL, *P*=0.557) although with no statistical difference. Curcumin supplementation indeed improved the lipid metabolism partially in the patients with T2DM from our results. Especially, its effect on reducing plasma TG and TC may be of important clinical relevance. It is indicated that TC and TG-rich lipoproteins are causally linked with the risk of atherosclerotic CVD. Therefore, inhibiting the elevation of TG and TC levels may be an important therapeutic strategy particularly in statin-treated patients with a high residual cardiovascular risk [34]. Although the change of LDL-c and HDL-c was not statistically significant in our meta-analysis, the effect of curcumin on LDL-c/HDL-c and its potential clinical significance could not be neglected. LDL-c is the primary predictor of CVD risk in T2DM. Mounting evidence has shown that increased LDL-c and non-HDL-c levels and decreased HDL-c levels are associated with an increased risk of cardiovascular disease in patients with diabetes [35–37]. The results of the Cholesterol Treatment Trialists with nearly forty thousand subjects showed that the aggressive reduction of LDL-c (approximately a 19 mg/dL difference) led to a 15% decrease in major vascular events, a 13% reduction in coronary death, a 19% decrease in coronary revascularization, and a 16% decrease in strokes compared with the usual reduction of LDL-c [38]. It suggested that aggressive lowering of LDL-c levels could further reduce cardiovascular events. Although HDL-c is not recommended as a target for treatment according to the guidelines [39], it is well known that low HDL-c is a risk factor for CVD. In this meta-analysis, the mean difference of LDL-c reduction was 4.01 mg/dL (the lower limit of the confidence interval was 10.96 mg/dL) between curcumin and placebo control. The increase of HDL-c was also higher in the curcumin group, indicating that curcumin potentially had more clinical benefits compared with the placebo. It was found that the lipid-lowering effect of curcumin may be due to increased hydrolysis of circulating lipoproteins induced by elevation of lipoprotein lipase activity [15]. On the other hand, dyslipidemia-induced fat deposition could aggravate the dysfunction of pancreatic beta cells and inhibit insulin-stimulated glucose uptake and glycogen synthesis in diabetes [40,41]. The attenuation of dyslipidemia by curcumin supplementation could in turn improve the glucose metabolic status of T2DM patients, and multiple molecular targets including PPAR-*γ* [32,33], cholesteryl ester transfer protein [42], and lipoprotein lipase [15] who contribute to the beneficial effects of curcumin.

In our meta-analysis, significant heterogeneity was found in pooled analyses of TG, LDL-c, FBG, and Hb1Ac. A random-effects model was used to compensate for heterogeneity, and subgroup analysis was performed to identify the source of heterogeneity. It revealed that trial duration, curcumin dosage, and other therapy may contribute to the variation in pooled effects. We found that a higher dose of curcumin was more powerful in reducing plasma TG and FBG concentrations. The patients with coexisting other therapy also had a lower level of TG and FBG. With regard to the treatment duration, there was a greater effect of curcumin on FBG and HbA1c in longer studies (>8 weeks). It seems that a longer treatment duration is needed to achieve changes in glucose metabolism.

The strength of this study was that the patients in this meta-analysis were all T2DM, which made our study to be the first to evaluate the effect of curcumin on lipid and glucose metabolism in the specific T2DM target population. Furthermore, subgroup analyses were performed to stratify the factors that might affect the results. Given the potential influence on clinical practice, our study may provide guidance for the use of curcumin in a specific clinical population. However, this meta-analysis also had several limitations that should be considered. The main limitations were that the number of included studies was few and most of the studies had small sample sizes. The treatment duration for some studies was short (<2 months) and it may be insufficient to affect some metabolic parameters [23]. In addition, the eligible articles were inhomogeneous in some ways. Though a random-effects model was applied and subgroup analyses were performed, heterogeneity could still have an impact.

## 5. Conclusions

In conclusion, this meta-analysis provided evidence that curcumin has promising effects on the lipid profile and glycemic status in patients with T2DM. It indicated that curcumin might be a favorable therapeutic option for T2DM patients with mixed dyslipidemia. However, considering the limitations of our study, further large-scale multicenter RCTs are required to confirm the clinical improvement of curcumin.

## Figures and Tables

**Table 1:** Characteristics of the subjects included in the studies.

Author, year	Study design	Duration	Study group	*N*	Mean age (years)	Baseline values (mg/dl)					
						Triglycerides	TC	LDL-c	HDL-c	FBG	HbA_1c_ (%)
Adibian et al., 2019 [20]	RDP	10 weeks	Curcumin 1500 mg/d	21	58 ± 8	124 ± 36	167 ± 34	112 ± 31	30 ± 2	160 ± 35	11.3 ± 1.6
			Placebo	23	60 ± 7	126 ± 52	180 ± 47	125 ± 44	30 ± 2	144 ± 40.6	11.2 ± 1.3
Adab et al., 2019 [21]	RDP	8 weeks	Turmeric 2100 mg/d	39	54.76 ± 6.00	181.56 ± 79.79	148.85 ± 36.11	82.56 ± 20.99	38.79 ± 10.30	133.79 ± 25.60	7.06 ± 1.01
			Placebo	36	55.66 ± 8.64	164.05 ± 81.19	155.36 ± 36.27	86.61 ± 21.99	44.63 ± 10.66	129.91 ± 32.98	6.79 ± 1.08
Abbood, 2018 [22]	RP	12 weeks	Curcumin 1000 mg/d	16	46.76 ± 7.89	193.15 ± 59.36	214.18 ± 60.31	109.02 ± 12.76	46.01 ± 9.28	190.44 ± 51.48	8.0 ± 0.91
			Atorvastatin 20 mg/d	16		225.93 ± 135.56	262.5 ± 68.43	114.43 ± 53.74	53.74 ± 9.67	214.56 ± 57.96	8.19 ± 1.24
			Placebo	16		170.11 ± 73.54	223.07 ± 48.33	105.16 ± 39.43	38.27 ± 11.6	210.24 ± 65.52	8.35 ± 1.93
Panahi et al., 2017 [12]	RDP	12weeks	Curcuminoids 1000 mg/d	50	43 ± 8	229.78 ± 81.84	217.34 ± 41.60	169.16 ± 30.77	40.86 ± 5.41	163 ± 37	7.4 ± 0.7
			Placebo	50	41 ± 7	207.62 ± 54.63	231.04 ± 70.95	199.06 ± 54.49	39.46 ± 6.09	174 ± 32	7.5 ± 0.9
Rahimi et al., 2016 [11]	RDP	3 months	Nano-curcumin 80 mg/d	35	56.34 ± 11.17	109 (94.75) #	163.4 ± 33.94	96.57 ± 33.94	54.30 ± 14.02	135.5 ± 51.33	7.59 ± 1.74
			Placebo	35	60.95 ± 10.77	142 (97.50) #	162.4 ± 38.59	99.78 ± 30.33	60.35 ± 15.96	148.30 ± 76.41	7.49 ± 1.75
Maithili et al., 2015 [23]	RP	4 weeks	Turmeric 2 g/d + Met 1 g/d	30	47 ± 7.17	120.56 ± 37.1	184.6 ± 14.6	124.4 ± 17	36.02 ± 8.1	116 ± 23	7.9 ± 1.3
			Met 1 g/d	30	46.8 ± 6.1	127.3 ± 33.3	181.5 ± 22.3	121.7 ± 26.3	34.3 ± 7.1	111 ± 24	7.8 ± 0.5
Na et al., 2013 [15]	RDP	3 months	Curcuminoids 300 mg/d	50	55.42 ± 6.40	2.23 ± 0.53	6.11 ± 1.13	4.30 ± 1.20	1.37 ± 0.26	8.58 ± 2.66	7.77 ± 1.82
			Placebo	50	54.72 ± 8.34	2.19 ± 1.04	6.08 ± 1.24	4.32 ± 1.15	1.33 ± 0.28	8.41 ± 2.17	7.72 ± 2.12
Khajehdehi et al., 2011 [24]	RDP	2 months	Turmeric 1500 mg/d	20	52.9 ± 9.2	236.2 ± 146.5	214.2 ± 66.5	114.1 ± 34.6	43.8 ± 12.6	179.0 ± 65.5	
			Placebo	20	52.6 ± 9.7	220.4 ± 106.9	193.3 ± 45.7	108.3 ± 39.9	39.8 ± 9.5	169.54 ± 76.3	
Usharani et al., 2008 [14]	RDP	8 weeks	Curcuminoids 600 mg/d	23	55.52 ± 10.76	176.39 ± 27.61	195.0 ± 41.16	120.35 ± 42.13	38.78 ± 7.69	155.04 ± 17.94	8.04 ± 0.85
			Atorvastatin 10 mg/d	23	50.47 ± 10.35	182.26 ± 43.85	196.78 ± 35.28	123.50 ± 38.73	36.82 ± 5.45	161.21 ± 19.74	8.30 ± 0.86
			Placebo	21	49.75 ± 8.18	170.14 ± 47.54	196.95 ± 35.72	125.29 ± 34.94	36.38 ± 7.67	161.19 ± 19.97	7.82 ± 0.57

Values are expressed as mean ± SD. ^#^Values expressed as Median (IQR).

**Table 2:** Subgroup analysis about trial duration, standard curcumin dose, and other therapy.

Subgroup and variable	Trials number	Mean difference (mg/dL) 95% CI	P	P-h
TG				
Duration				
>8w	5	−17.473 (−37.464, 2.517)	0.087	0.008
≤8w	4	−21.614 (−56.314, 13.086)	0.222	≤0.001
Standard curcumin dose				
>100 mg/day	6	−28.944 (−49.620, −8.268)	0.006	≤0.001
≤100 mg/day	3	−18.974(−36.473, −1.474)	0.173	0.698
Other therapy				
Y	5	−39.449 (−65.370, −13.527)	0.003	0.004
N	4	2.275 (−6.819, 11.369)	0.624	0.382

LDL-c				
Duration				
>8w	5	−2.873 (−15.134, 9.388)	0.646	0.018
≤8w	4	−5.990 (−13.292, 1.312)	0.108	0.295
Standard curcumin dose				
>100 mg/day	6	−4.118 (−14.116, 5.880)	0.419	0.26
≤100 mg/day	3	−3.729 (−14.089, 6.630)	0.480	0.212
Other therapy				
Y	5	−9.260 (−16.271, −2.248)	0.010	0.356
N	4	1.529 (−9.945, 13.003)	0.794	0.38

HbA1c (%)				
Duration				
>8w	5	−0.749 (−1.059, −0.439)	≤0.001	0.183
≤8w	3	−0.170 (-0.432, 0.092)	0.205	0.304
Standard curcumin dose				
>100 mg/day	6	−0.495 (−0.777, −0.213)	0.001	0.032
≤100 mg/day	2	−0.693 (−1.711, 0.325)	0.182	0.005
Other therapy				
Y	4	−0.483 (−0.735, −0.231)	≤0.001	0.337
N	4	−0.551 (−0.983, −0.120)	0.012	0.001

FBG				
Duration				
>8w	5	−10.893 (−21.149, −0.636)	0.037	0.054
≤8w	4	−7.620 (−14.845, −0.395)	0.039	0.234
Standard curcumin dose				
>100 mg/day	6	−9.798 (−16.375, −3.221)	0.004	0.089
≤100 mg/day	3	−2.000 (−20.593, 16.592)	0.833	0.134
Other therapy				
Y	5	−15.185 (−24.819, −5.550)	0.002	0.251
N	4	−5.757 (−10.871, −0.643)	0.027	0.262

## Data Availability

The data used to support the findings of this study are included within the article.

## Abbreviations

RDP: Randomized double-blind parallel
RP: Randomized parallel
Met: Metformin
TC: Total cholesterol
LDL-c: Low density lipoprotein cholesterol
HDL-c: High density lipoprotein cholesterol
FBG: Fasting blood glucose
N: Number of participants.
